# Sex Differences in Cardiac Involvement in Adults With Myotonic Dystrophy Type 1: A Multicenter Study

**DOI:** 10.1016/j.jacadv.2026.102771

**Published:** 2026-04-28

**Authors:** Alberto Alen, Carmen Muñoz, Marc Soriano-Amores, Fernando de Frutos, Néstor Báez-Ferrer, María Luisa Peña-Peña, Eduardo Villacorta, Tomas Ripoll-Vera, Esther Zorio, Aaron Martínez- Gimeno, José Bermúdez-Jiménez, Javier Limeres, Coloma Tiron, Esteban Martínez-Álvarez, Eva Cabrera-Romero, Pablo García-Pavía, María Angeles Espinosa, Jesús Piqueras, Soledad García-Hernández, Julián Palomino-Doza, Carlos Moliner-Abós, German Moris, Lidia María Carrillo-Mora, Petros Syrris, Rut Alvarez-Velasco, Juan Ramón Gimeno, Rebeca Lorca

**Affiliations:** a Área del Corazón, Hospital Universitario Central Asturias, Oviedo, Spain; b Unidad CSUR/ERN de Cardiopatías Familiares, Hospital Universitario Virgen de la Arrixaca, Murcia, Spain; c Cardiology Department, Hospital de la Santa Creu i Sant Pau, IIb-SantPau, CIBERCV, Universitat Autónoma de Barcelona, Barcelona, Spain; d Heart Failure and Inherited Cardiac Diseases Unit, Department of Cardiology, Hospital Universitari de Bellvitge, L’Hospitalet de Llobregat, Barcelona, Spain; e Bioheart Group, Cardiovascular, Respiratory and Systemic Diseases and Cellular Aging Program, Institut d’Investigació Biomèdica de Bellvitge (IDIBELL), L’Hospitalet de Llobregat, Barcelona, Spain; f Cardiology Department, Hospital Universitario de Canarias, Santa Cruz de Tenerife, Spain; g Instituto de Investigación Sanitaria de Canarias, Tenerife, Spain; h Unidad de Cardiopatías Familiares, Servicio de Cardiología, Hospital Universitario Virgen del Rocío, Sevilla, Spain; i Inherited Heart Disease Unit Cardiology Department, Hospital Universitario Álvaro Cunqueiro, Vigo, Spain; j Inherited Heart Disease Unit, Hospital Universitario Son Llatzer & IdISBa, Palma de Mallorca, Spain; k CIBER Cardiovascular, Instituto de Salud Carlos III, Madrid, Spain; l Department of Cardiology, Hospital Universitario y Politécnico La Fe, Valencia, Spain; m Instituto de Investigación Sanitaria La Fe (CAFAMUSME Research Gruop), Valencia, Spain; n Departamento de Medicina, Universidad de Valencia, Valencia, Spain; o Department of Neurology, Hospital Universitario y Politécnico La Fe, Valencia, Spain; p Department of Cardiology, Virgen de las Nieves University Hospital, Instituto de Investigación Biosanitaria (ibs.GRANADA), Granada, Spain; q Inherited Cardiac Disease Unit, Servicio de Cardiología, Vall d’Hebron University Hospital, Vall d’Hebron Institut de Recerca (VHIR), Universitat Autonoma de Barcelona, Barcelona, Spain; r Inherited Cardiac Diseases Unit, Department of Cardiology, Hospital Universitari Dr Josep Trueta, Girona, Spain; s Centro de Investigación Biomédica en Red de Enfermedades Cardiovasculares, Madrid, Spain; t Inherited Cardiac Disease Unit, Hospital Universitario de A Coruña (HUAC), Servizo Galego de Saúde (SERGAS), A Coruña, Spain; u Instituto de Investigación Biomédica de A Coruña (INIBIC), Universidade da Coruña (UDC), A Coruña, Spain; v Heart Failure and Inherited Cardiac Diseases Unit, Department of Cardiology, Hospital Universitario Puerta de Hierro, IDIPHISA, Madrid, Spain; w European Reference Network for Rare and Low Prevalence Complex Diseases of the Heart: ERN GUARD-Heart, Amsterdam, The Netherlands; x Centro Nacional de Investigaciones Cardiovasculares (CNIC), Madrid, Spain; y Universidad Francisco de Vitoria (UFV), Pozuelo de Alarcón, Spain; z Department of Cardiology, Hospital General Universitario Gregorio Marañón, Madrid, Spain; aa Instituto de Investigación Sanitaria Gregorio Marañón, Madrid, Spain; ab Facultad de Medicina, Universidad Complutense, Madrid, Spain; ac Unidad de Cardiopatías Familiares e Insuficiencia Cardiaca, Hospital General Universitario de Ciudad Real, Ciudad Real, Spain; ad Facultad de Medicina, Universidad de Castilla La Mancha, Castilla la Mancha, Spain; ae Instituto de Investigación Biomédica de Castilla La Mancha (IDISCAM), Castilla La Mancha, Spain; af San Cecilio University Hospital, Granada, Spain; ag Health in Code S.L., A Coruña, Spain; ah Inherited Cardiac disease Unit, Hospital Universitario 12 de Octubre, Instituto de Investigación i+12, Madrid, Spain; ai Instituto de Investigación Sanitaria del Principado de Asturias (ISPA), Oviedo, Spain; aj Universidad de Oviedo, Oviedo, Spain; ak Neurology Department, Hospital Universitario Central Asturias, Oviedo, Spain; al Unidad CSUR/ERN de Cardiopatías Familiares, Hospital Universitario Virgen de la Arrixaca, Murcia, Spain; am Centre for Heart Muscle Disease, UCL Institute of Cardiovascular Science, London, UK; an Departamento de Medicina Interna, Universidad de Murcia, Murcia, Spain; ao Redes de Investigación Cooperativa Orientadas a Resultados en Salud (RICORs), Madrid, Spain

**Keywords:** arrythmia, conduction abnormalities, ICD, myotonic dystrophy, pacemakers, sex differences

## Abstract

**Background:**

Myotonic dystrophy type 1 (DM1) is associated with progressive cardiac conduction abnormalities (CCAs) and arrhythmias. Although genetic anticipation and CTG repeat length influence disease severity, sex-related differences in cardiac involvement remain underexplored.

**Objectives:**

We aimed to investigate sex differences in cardiac outcomes in a large cohort of adult DM1 patients and assess whether these differences persist after adjustment for generational effects.

**Methods:**

Clinical data from 549 DM1 patients followed at 16 inherited cardiac disease clinics in Spain were analyzed. The primary endpoint was a composite of lifetime CCA development, device implantation, major ventricular arrhythmias, and cardiac syncope. Secondary endpoints included overall survival, atrial fibrillation (AF), and device implantation. Birth cohort (representing generation) was used as a surrogate for underlying genetic burden, as later generations tend to accumulate larger CTG expansions.

**Results:**

When compared to females, males had a higher cumulative incidence of the primary endpoint (subdistribution HR [sHR]: 1.50; 95% CI: 1.12-2.00; *P* = 0.006), device implantation (sHR: 1.49; 95% CI: 1.04-2.14; *P* = 0.029) and AF (sHR: 2.19; 95% CI: 1.36-3.53; *P* = 0.001). Males more commonly presented with first-degree atrioventricular block compared to females (46.7% vs 37.9%; *P* = 0.037), but there was no significant difference in second- or third-degree atrioventricular block or QRS ≥120 ms. Sex differences in primary endpoint, device implantation, and AF persisted after generational adjustment (all *P* < 0.05). Overall survival was similar between sexes (*P* = 0.472).

**Conclusions:**

In this large DM1 cohort, male sex was associated with a higher cumulative incidence of earlier CCA, device implantantion, and AF, beyond generational/genetic effects. These findings support sex-informed risk stratification.

Myotonic dystrophy type 1 (DM1) (Steinert’s disease; OMIM 160900) is the most prevalent adult-onset muscular dystrophy.^1^ This autosomal dominant disorder arises from an unstable CTG trinucleotide repeat expansion in the myotonic dystrophy protein kinase gene, and manifests with a broad spectrum of neuromuscular and multisystem involvement, including progressive muscle weakness, myotonia, cataracts, respiratory insufficiency, endocrine disturbances, cognitive impairment, and cardiac disease.^2^, ^3^, ^4^ The size of the CTG expansion generally correlates with disease severity, with mildly affected individuals typically carrying 50 to 100 repeats, and more severely affected patients harboring 100 to 1,000 repeats.^2^ Across generations, CTG repeats tend to expand, giving rise to genetic anticipation, characterized by earlier onset and more severe manifestations in successive generations.^5^^,^^6^

Cardiac involvement in DM1 is a major determinant of morbidity and mortality, particularly due to conduction abnormalities and arrhythmias, which may progress insidiously and are often independent of peripheral muscle involvement.^7^^,^^8^ Current DM1 registries face persistent challenges in elucidating the nature and predictors of cardiac involvement, a gap that is particularly critical as research advances toward therapeutic development.^9^

Large DM1 cohorts have highlighted the impact of age at diagnosis, generational cohort, and CTG repeat length on the timing and severity of cardiac conduction disease, and these factors are increasingly recognized as key determinants of cardiac risk. In this context, birth cohort (representing generation) can be used as a pragmatic surrogate for underlying genetic burden, as later generations tend to accumulate larger CTG expansions and show earlier and more severe phenotypes.^10^ However, sex-specific differences in DM1 manifestations have been less thoroughly explored. Previous studies suggest that male may experience worse survival and neuromuscular and cardiopulmonary prognosis,^9^ with more severe myotonia, cardiac involvement, respiratory impairment, and muscle weakness, whereas female more frequently present cataracts, dysphagia, gastrointestinal dysfunction, incontinence, thyroid disorders, and obesity.^9^^,^^11^, ^12^, ^13^, ^14^ However, robust data on sex-specific differences in cardiac conduction abnormalities(CCAs), arrhythmic events, and device implantation—particularly after accounting for generational and genetic factors—remain limited. A better understanding of these differences is clinically relevant, as it may support sex-specific surveillance strategies and tailored risk stratification.

In this study, using a large, nationwide DM1 cohort, the aim was to investigate sex-specific differences in cardiac involvement, focusing on the occurrence and timing of CCAs, arrhythmias, and device implantation. To approximate underlying genetic burden, patients were additionally stratified by birth cohort into 3 generations, previously shown to mirror the progressive expansion of CTG repeats and the anticipation phenomenon. This design allowed assessment of whether sex-related differences in cardiac outcomes persist after adjustment for generational effects as a surrogate for genetic severity.

## Methods

### Study population

This analysis used the identical multicenter cohort of 549 adult DM1 patients (mean age at diagnosis 33.6 ± 16.2 years; 48.1% female) with definite diagnosis based on clinical/neurophysiological features and/or genetic CTG confirmation, followed at the same 16 Spanish inherited cardiac disease units/cardiomyopathy clinics as in *Lorca* et al.^10^ They were followed at 16 inherited cardiac disease units and cardiomyopathy clinics in Spain. Exclusion criteria were identical: congenital and childhood-onset forms, those without cardiovascular evaluation/follow-up data, or registry refusers.

The multicenter study was approved by the Local Ethics Committee (CEImPA 2023.206) and conformed to the 1964 Declaration of Helsinki principles and its later amendments. The authors from each participating center guarantee the integrity of the data.

### Data acquisition

Data collection mirrored the standardized, anonymized process detailed in Lorca et al,^10^ including demographics (age, sex, diagnosis reason, and family history), neuromuscular status (muscular involvement and wheelchair use), cardiovascular tests (electrocardiogram [ECG], 24-h Holter, echocardiogram, and electrophysiological studies at baseline/follow-up/last visit), events, and available CTG repeats. Atrial fibrillation (AF) detection included any validated method according to guidelines (ECG, Holter monitoring, or implantable devices).^15^ Device implantation—either pacemaker (PM) or implantable cardioverter-defibrillator (ICD)—as well as the indication and timing of electrophysiological studies, were left to clinician judgment according to local clinical practice at each participating center and in line with contemporary guidelines.^5^^,^^6^ Decisions regarding device therapy similarly involved shared decision-making, ensuring that patient values and preferences were explicitly considered when discussing potential benefits and risks.

### Study endpoints

The primary endpoint was to compare a composite of adverse cardiovascular events between sex, including CCAs development over the lifetime, device implantation (PM and/or ICD), major ventricular arrhythmias (MVA), and/or cardiogenic syncope.^10^

CCAs were considered when at least 1 of the following features was present: 1) first-degree atrioventricular block (AVB) (AVB-I) defined by PR interval of 200 ms plus QRS duration of 120 ms or more, including right and left bundle branch blocks; 2) HV interval equal to or longer than 70 ms in electrophysiological evaluation; and 3) second-degree or third-degree AVB (AVB-II or AVB-III).^10^

MVA included documented sudden cardiac death, aborted sudden cardiac death, sustained ventricular tachycardia and ventricular fibrillation.^10^

Patients were stratified by the same generations (1920-1965 [1st], 1966 to 1990 [2nd], and 1991 to 2015 [3rd]) as previously.^10^

Secondary endpoints were a comparison of overall survival, device implantation and AF over lifetime. AVB-I, AVB-II, AVB-III, and QRS prolongation were also evaluated separately.

### Statistical analyses

Categorical variables are presented as counts and percentages, and continuous variables as mean ± SD or median with IQR, as appropriate according to their distribution. Between-group comparisons were performed using the chi-square test or Fisher exact test for categorical variables, and the Student’s t-test or Mann-Whitney U test for continuous variables, as appropriate.

Time-to-event analyses were conducted to evaluate the association between sex (male vs female) and study outcomes. Kaplan-Meier methods were initially used to estimate event-free survival, and groups were compared using the log-rank test.

Given the potential competing risk of death, cumulative incidence functions were estimated for the primary endpoint and key secondary outcomes (AF, device implantation, and AVB-I), and comparisons between groups were performed using the Gray test. Subdistribution hazard models according to Fine and Gray were used to assess the association between sex and these outcomes, accounting for death as a competing event. Subdistribution HR (sHR) >1 indicates a higher subdistribution hazard (cumulative incidence) of the event.

Multivariable analyses were performed using Cox proportional hazards regression models to estimate HRs and 95% CIs for outcomes not analyzed under competing risk assumptions. Cox HR > 1 indicates higher cause-specific hazard. Multivariable Fine-Gray and Cox analyses were adjusted for generation (1920-1965, 1966-1990, and 1991-2015) as a surrogate of genetic burden. Proportional hazards/subdistribution assumptions verified using Schoenfeld residuals.

Follow-up time and number of visits are presented as median (IQR) due to their expected non-normal distribution. Missing data were handled using complete-case analysis.

All tests were 2-sided, and a *P* value <0.05 was considered statistically significant. Statistical analyses were performed using Stata/IC (version 15.1, StataCorp).

## Results

### Study population

Clinical information on 549 DM1 patients was submitted from 16 Spanish hospitals. The general characteristics of the baseline cohort displayed divided by male and female is presented in Table 1. Nearly half of the patients were females (48.1%). The median follow-up duration was 11.10 years (IQR: 7.05-14.47), during which a median of 5 visits including ECGs (IQR: 3-8) were performed. There were no significant differences between groups in terms of age at diagnosis, muscular affectation/need of wheelchair or in the number of CTG triplet expansion (Table 1). Although family screening was the main reason for DM1 diagnosis, when compared to males, females were significantly more often diagnosed due to symptoms (Table 1).

**Table 1:** Clinical Characteristics of the Cohort DM1 Patients, Displayed by Sex

	Total (N = 549)	Male (n = 285)	Female (n = 264)	*P* Value
Age at diagnosis, y (±SD)	33.6 (±16.2)	32.8 (±17.0)	34.6 (±15.1)	0.193
Reason for diagnosis				0.018^a^
Family screening	309 (56.3%)	176 (61.8%)	133 (50.4%)	
Symptoms	226 (41.2%)	104 (36.5%)	122 (46.2%)	
Casual	14 (2.6%)	5 (1.8%)	9 (3.41%)	
Muscular affectation	487 (88.7%)	257 (90.2%)	230 (87.1%)	0.259
Wheelchair	53 (9.7%)	34 (11.9%)	19 (7.2%)	0.061
Active worker	217 (51.3%)	109 (50.9%)	108 (51.7%)	0.879
CTG-expansion	234.95 (±388.8)	261.95 (±465.0)	207.96 (±292.8)	0.251
Deaths (all-cause of death)	66 (12.00%)	37 (13.0%)	29 (11.0%)	0.472
Mean age at death	59.06 (±10.8)	58,08 (±11.4)	61.6 (±8.9)	
CV deaths/deaths	12 (18.18%)	8 (21.6%)	4 (13.8%)	0.413
Mean age at CV death	58.40 (±13.5)	54.9 (±13.4)	65.2 (±13.4)	
Generations				0.115
1920-1965	155 (28.23%)	84 (31.82%)	71 (24.91%)	
1966-1990	322 (58.65%)	147 (55.68%)	175 (61.40%)	
1991-2015	72 (13.11%)	33 (12.50%)	39 (13.68%)	

Values are mean (±SD) or n (%). *P* values compare male and female. Cardiovascular (CV) deaths refer to deaths attributed to cardiovascular causes. CTG expansion indicates the number of CTG trinucleotide repeats.

DM1 = myotonic dystrophy type 1.

a Statistical significance was defined as *P* < 0.05.

During lifetime, the cumulative incidence of the primary composite endpoint was higher in males compared with females with DM1, after accounting for death as a competing event (Central Illustration) (sHR: 1.50; 95% CI: 1.12-2.00; *P* = 0.006). The mean time from baseline to the primary endpoint was 3.46 years (SD: 7.33).

**Central Illustration:**
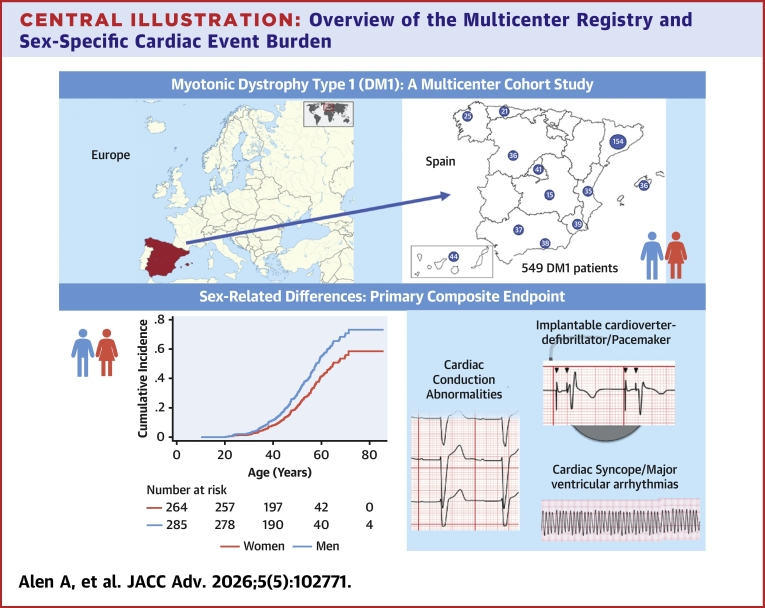
Overview of the Multicenter Registry and Sex-Specific Cardiac Event Burden Top panel: map of Spain and Europe showing the location of the 16 participating centers and the number of patients contributed by each site. Bottom panel: incidence of the primary composite endpoint (development of significant cardiac conduction abnormalities, device implantation, and cardiac syncope/major ventricular arrhythmias).

**Figure 1:**
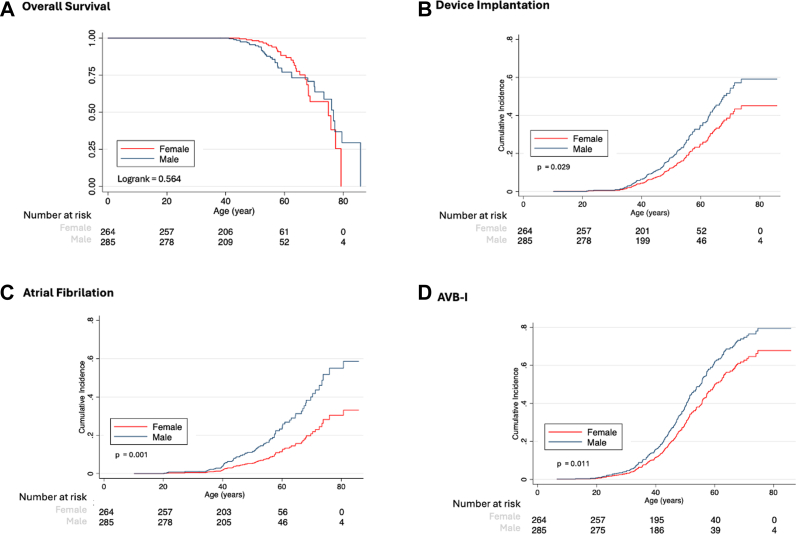
Sex-Based Cardiac Outcomes in Myotonic Dystrophy Type 1 (A) Kaplan–Meier curves show overall survival. Cumulative incidence functions accounting for death as a competing risk are shown for (B) device implantation, (C) atrial fibrillation, and (D) first-degree atrioventricular block (AVB-I). Curves are stratified by sex. Numbers at risk are displayed below each pane.

However, overall survival did not differ significantly between male and female patients (Figure 1A). The cumulative incidence of device implantation was higher in males (Figure 1B) (sHR: 1.49; 95% CI: 1.04-2.14; *P* = 0.029). Likewise, the cumulative incidence of AF (Figure 1C) (sHR: 2.19; 95% CI: 1.36-3.53; *P* = 0.001) and AVB-I (Figure 1D) (sHR: 1.40; 95% CI: 1.08-1.81; *P* = 0.011) was higher in males. No statistically significant differences were observed in QRS prolongation (Cox HR: 1.33; 95% CI: 0.98-1.80; *P* = 0.065), CCA (HR: 1.38; 95% CI: 0.99-1.92; *P* = 0.059), and syncope or MVA (HR: 1.66; 95% CI: 0.75-3.67; *P* = 0.207).

Multivariable Fine-Gray competing risk analysis for the primary endpoint confirmed male sex as an independent predictor (sHR: 1.39; 95% CI: 1.02-1.88; *P* = 0.034) after adjustment for generation, reason for diagnosis and wheelchair use (Table 2). Third generation showed higher risk vs reference (sHR: 3.36; 95% CI: 2.4-4.67; *P* < 0.001), whereas wheelchair use (sHR: 1.62; 95% CI: 1.05-2.5; *P* = 0.028) and symptomatic diagnosis were also independently associated (sHR: 0.7; 95% CI: 0.52-0.94; *P* = 0.019). These results confirm that sex differences persist after comprehensive multivariable adjustment beyond generational/genetic effects.

**Table 2:** Multivariable Fine-Gray Competing Risk Analysis (Primary Endpoint)

Covariate	sHR	Robust SE	z	*P* Value	95% CI
Sex (male)	1.39	0.22	2.12	0.034	1.02-1.88
Generations	3.36	0.56	7.2	0.000	2.40-4.67
Reason for diagnosis	0.70	0.12	−2.34	0.019	0.52-0.94
Wheelchair	1.62	0.36	2.19	0.028	1.05-2.50

Multivariable competing risk analysis using the Fine-Gray model for the primary endpoint. Male sex (sHR = 1.39; *P* = 0.034), generation (sHR = 3.36; *P* < 0.001), and wheelchair use (sHR = 1.62; *P* = 0.028) are associated with a higher risk of the event.

sHR = subdistribution HRz.

Most patients with DM1 underwent PM implantation (19.7%), whereas ICD implantation was less frequent (4.0%) (Table 3). The prevalence of AF was higher in male patients (17.9% vs 9.1%; *P* = 0.003) (Table 3). Of the 75 AF cases, the detection method was available in 74: 59 (79.7%) were identified by ECG or Holter monitoring and 15 (20.3%) through implanted devices. Device-detected AF showed a similar distribution between sexes (male 18.0% vs female 25.0%; *P* = 0.543).

**Table 3:** Study Endpoints and Cardiac Conduction Abnormalities, Displayed by Sex

	Total (N = 549)	Male (n = 285)	Female, (n = 264)	*P* Value
Results				
Primary endpoint	182 (33.15%)	106 (37.19%)	76 (28.79%)	0.037^a^
Secondary endpoint: Overall survival	66 (12.02%)	37 (12.98%)	29 (10.98%)	0.472
Secondary endpoint: Atrial fibrillation	75 (13.66%)	51 (17.9%)	24 (9.1%)	0.003^a^
Secondary endpoint: Device implantation				
Pacemaker implantation	108 (19.7%)	61 (21.4%)	47 (17.8%)	0.289
Implantable cardioverter defibrillator	22 (4.0%)	17 (6.0%)	5 (1.9%)	0.015^a^
Cardiac conduction abnormalities				
AVB-I	233 (42.4%)	133 (46.7%)	100 (37.9%)	0.037^a^
AVB-II	30 (5.5%)	18 (6.3%)	12 (4.5%)	0.362
AVB-III	10 (1.8%)	5 (1.9%)	5 (1.8%)	0.903
QRS ≥120 mseg	170 (31.0%)	95 (33.3%)	75 (28.4%)	0.213

Values are n (%). *P* values compare male and female. The primary endpoint reflects the composite outcome defined in the Methods section. Secondary endpoints include overall survival, atrial fibrillation, and device implantation. Cardiac conduction abnormalities were assessed by serial electrocardiograms.

AVB-I = first-degree atrioventricular block; AVB-II = second-degree atrioventricular block; AVB-III = third-degree atrioventricular block.

a Statistical significance was defined as *P* < 0.05.

No statistically significant differences were observed between sexes in the incidence of AVB-II, AVB-III, or QRS prolongation ≥120 ms (Table 3). However, the incidence of AVB-I was higher in male patients.

Indications for PM implantation (conduction disturbance on ECG, prolonged HV interval, 2:1 AV block, AVB-III, syncope) and for ICD implantation (primary vs secondary prevention) were similar between groups (*P* = 0.846 and *P* = 0.055), suggesting that differences in device indications are unlikely to account for the observed outcome differences (Table 4).

**Table 4:** Indications for Pacemaker and Implantable Cardioverter-Defibrillator Implantation

	Indications for PM Implantation			
	Total (N = 108)	Male (n = 61)	Female (n = 47)	*P* Value
Conduction disturbance in ECG	12 (11.11%)	7 (11.11%)	5 (10.64%)	0.846
Prolonged HV interval	10 (9.26%)	8 (13.11%)	2 (4.26%)	
2:1 AV block	35 (32.41%)	16 (26.23%)	19 (40.43%)	
Third-degree AV block	40 (37.04%)	22 (36.07%)	18 (38.30%)	
Syncope	11 (10.19%)	8 (13.11%)	3 (6.38%)	

	**Indications for ICD Implantation**			
	**All (N = 22)**	**Male (n = 17)**	**Female (n = 5)**	***P* Value**

Primary prevention	17 (77.27%)	15 (88.24%)	2 (40.0%)	0.055
Secondary prevention	5 (60.0%)	2 (11.76%)	3 (60%)	

Values are number of patients and stratified by sex. Pacemaker (PM) indications included conduction disturbances on electrocardiogram (ECG), prolonged His–ventricle (HV) interval, 2:1 atrioventricular (AV) block, third-degree AV block, and syncope. ICD implantation was performed for primary or secondary prevention of sudden cardiac death.

The associations between sex and the primary endpoint remained consistent after adjustment for generation at diagnosis, supporting that these findings are independent of genetic anticipation.

## Discussion

In this large cohort of 549 adults with DM1, males showed higher cumulative incidence of the primary composite endpoint (sHR: 1.50; 95% CI: 1.12-2.00; *P* = 0.006), device implantation (sHR: 1.49; 95% CI: 1.04-2.14; *P* = 0.029), AVB-I (sHR: 1.40; 95% CI: 1.08-1.81; *P* = 0.011), and AF (sHR: 2.19; 95% CI: 1.36-3.53; *P* = 0.001), after accounting for competing risk of death (Central Illustration, Figure 1). No sex differences were observed in overall survival (*P* = 0.472), QRS prolongation (sHR: 1.33; *P* = 0.065), CCA (HR: 1.38; *P* = 0.059), or syncope/MVA (HR: 1.66; *P* = 0.207). Baseline clinical profiles and CTG repeat lengths between male and female were comparable. These patterns confirm higher conduction disease and AF burden in males, driving greater device needs without increased mortality or ventricular risk.

A key strength of the present analysis is multivariable adjustment for generational cohort, used as a surrogate for genetic burden and genetic anticipation. Prior analysis^10^ showed that later generations, younger diagnosis, and larger CTG expansions were associated with earlier/severe cardiac events (eg, primary endpoint HR 35.3, 3rd vs 1st generation). Here, male sex effects on primary endpoint, devices, AVB-I, and AF persisted after generational adjustment (all *P* < 0.05), despite similar CTG lengths between sexes (*P* = 0.251) (Table 1). This isolate sex as an independent modifier beyond genetic burden, implicating hormonal, biological, or conduction-specific vulnerabilities.

Large DM1 registries^7^^,^^16^, ^17^, ^18^, ^19^, ^20^, ^21^, ^22^, ^23^, ^24^, ^25^ established conduction disease dominance, but sex-stratified analyses remain scarce. Notably, large registries such as Wahbi et al (n = 1,388),^18^ Chong-Nguyen et al (n = 855),^22^ and Groh et al (n = 406)^23^ have established conduction disease as the dominant cardiac feature, yet few incorporated sex-stratified analyses. Our findings align with Dogan et al.^11^^,^^12^ (higher male conduction hospitalizations), Zhong et al^13^ (4.8-year earlier male onset), Garibaldi et al^14^ (male conduction risk independent of genetics), Cudia et al^26^ (male arrhythmic risk OR: 2.17); and others.^9^^,^^27^

It is known that the prevalence of AF in the general population is around 2% to 4%, with significant differences between sexes and a progressive increase in incidence with age, reaching a prevalence of 1 in 3 from the age of 55 onwards.^28^^,^^29^ Notably, AF prevalence in this cohort (13.7%) exceeded benchmarks like the 3,677-patient review (10.9%; male-favoring^25^), aligning with a 96-patient study showing female protection (HR: 0.08) vs younger age (HR: 0.95/year) and CTG length (HR: 1.09/50 repeats; AF 28%, atrial flutter 8%, overlap 6/37).^30^ Device rates (PM 19.7%, ICD 4%) also surpassed literature reports (4.1%, 1.1%^17^), again with a male ICD predominance (*P* = 0.015). These findings are consistent with the greater burden of conduction disease and AF observed in male, more pronounced than in the general population. They extend previous evidence by demonstrating these differences in a large, well-characterized cohort and further support a sex-specific vulnerability of the conduction system in DM1. There is a well-described sex disparity in ICD/PMs implantation rates, not fully explained by epidemiological differences in the prevalence of cardiomyopathies, which could imply undertreatment of female.^31^^,^^32^

Several mechanisms may explain these differences. Maternal inheritance drives severe expansions via female germline instability,^3^^,^^33^ yielding "classic" male phenotypes (myotonia, cardiac/respiratory issues) vs female extramuscular features, enabling occult transmission.^12^ Sex-dependent oxidative stress^34^ or hormonal disparities^11^ could further amplify male cardiac risk, as generational/CTG adjustment affirms.

From a clinical perspective, these results have several implications. First, male sex in DM1 emerges as a readily available risk marker for intensified surveillance, earlier electrophysiological study,^35^ or device requirement, even when generational and genetic context are considered. Equivalent female vigilance remains essential given substantial morbidity. These findings expose important limitations of current risk-stratification algorithms in DM1, which do not yet incorporate sex, generational cohort, or CTG repeat length as formal risk-modifying factors. The present findings argue for future guideline updates and prospective studies to evaluate whether sex- and generation-informed algorithms can better identify high-risk DM1 patients and optimize timing of device therapy and arrhythmia prevention.

Further research, including prospective trials, validating sex-generation-informed algorithms for high-risk patient identification and optimized device/arrhythmia management are imperative. Mechanistic studies incorporating detailed genetics (somatic instability), hormonal profiling, longitudinal clinical, and electrophysiological phenotyping will elucidate underlying pathways and may refine precision strategies in DM1.

### Study limitations

This retrospective analysis shares limitations of the source cohort.^10^ Detailed information on center-specific protocols (including standardized criteria for electrophysiology study) and on cardiovascular and noncardiovascular comorbidities was not systematically available, which may have introduced heterogeneity in clinical practice, selection bias, and residual confounding. In addition, the multicenter design spanning decades may have generated variability in screening intensity, device implantation thresholds, and genetic testing. Although this is the largest national DM1 cohort described to date, small numbers of events in some subgroups limit power for rare outcomes and preclude full generalization. CTG repeat length was only available in approximately 50% of patients and somatic mosaicism was not systematically assessed, potentially underestimating genetic effects. Finally, no central adjudication of events or syncope was performed, and unmeasured confounders (eg, surveillance bias) may have influenced timing differences, underscoring the need for prospective validation in diverse populations. Finally, AF detection relied on any available method (ECG, Holter, or device detection), with device-detected cases (20.5%) potentially introducing detection bias given the higher implantation rate in male.

## Conclusions

In this large DM1 cohort, male sex was associated with higher cumulative incidence of earlier CCAs, device implantation needs, and AF—persisting beyond generational/genetic effects.^10^ These findings advocate sex- and generation-informed risk stratification, intensified monitoring in males, and integration into DM1 guidelines alongside generational factors for optimized cardiac surveillance.

## Funding support and author disclosures

This research was funded by Health Institute Carlos III, grant number PI22/00705, INT23/00054, and 10.13039/100009664Sociedad Española de Cardiología, grant code “SECARIT-INV-MUL 24/01”. The authors have reported that they have no relationships relevant to the contents of this paper to disclose.

Perspectives **COMPETENCY IN MEDICAL KNOWLEDGE:** In DM1, male sex showed higher cumulative incidence of earlier CCAs, AF, and device implantation, beyond generational effects.

**COMPETENCY IN PATIENT CARE AND PROCEDURAL SKILLS:** Male patients with DM1 may require earlier rhythm surveillance and proactive electrophysiologic evaluation to facilitate timely device implantation and arrhythmia prevention.

**COMPETENCY SYSTEMS-BASED PRACTICE:** Incorporating sex into electrophysiology risk stratification may improve selection and timing of monitoring strategies and device therapy in inherited arrhythmia programs.

**TRANSLATIONAL OUTLOOK:** Further research is needed to define the electrophysiologic mechanisms underlying sex-related differences in DM1 and to prospectively test sex-specific thresholds for rhythm monitoring and device implantation. Translational efforts should focus on integrating sex-based risk models into electrophysiology guidelines and clinical decision pathways.
